# Commentary: The Many Faces of COVID-19 at a Glance: A University Hospital Multidisciplinary Account From Milan, Italy

**DOI:** 10.3389/fpubh.2021.748263

**Published:** 2021-09-14

**Authors:** Ahmed Al Khalil, Marissa Absi, Nayaar Islam, Sanam Ebrahimzadeh, Matthew D. F. McInnes

**Affiliations:** ^1^Department of Radiology, Faculty of Medicine, University of Ottawa, Ottawa, ON, Canada; ^2^Department of Radiology, Clinical Epidemiology Program, Ottawa Hospital Research Institute, Ottawa, ON, Canada; ^3^Department of Radiology, Clinical Epidemiology Program, Ottawa Hospital Research Institute, University of Ottawa, Ottawa, ON, Canada

**Keywords:** COVID-19, diagnostic accuracy, chest CT, evidence-based medicine, living systematic review

We thank Priori et al. for their case study reporting the clinical, pathological, and radiological findings they observed in COVID-19 patients at their teaching hospital in Milan, Italy (1). The authors stated that chest CT has low specificity for COVID-19 infection in suspected patients and therefore cannot distinguish SARS-CoV2 infection from other respiratory diseases (1). This conclusion is based on the first version of a “Living” Cochrane Systematic Review on the diagnostic accuracy of imaging tests for COVID-19, published in September 2020, which identified that chest CT had a specificity of 18.1% [95% confidence interval (95% CI) 3.71–55.8] and sensitivity of 86.2% (95% CI 71.9–93.8) (2).

The “Living” Systematic Review strives to stay updated as new evidence emerges in this continuously evolving field of research. The second version of this review was published in November 2020 (3), and the third, most recent version, was published in March 2021 (4). In the latest version, the specificity of chest CT was 80.0% (95% CI 74.9–84.3) and the sensitivity was 87.9% (95% CI 84.6–90.6); the pooled estimates were drawn from 41 studies and 16,133 participants (4). The substantial rise in specificity can be possibly explained by the use of scoring systems (such as CO-RADS), which provide better definitions for index test positivity. Another explanation may be higher quality studies emerging later in the pandemic, benefitting from improved knowledge about COVID-19 (3). Additionally, the latest version includes an evaluation of the diagnostic accuracy of X-ray and ultrasound in COVID-19 infection (4). Our hope is that authors continue to prioritize transparent reporting and methodological rigor in future studies. The team conducting this Cochrane review will continuously aim to provide the most up to date evidence on the diagnostic accuracy of these imaging modalities.

## Author Contributions

MA conceived and presented the idea. AA put the data together and presented it coherently into a draft manuscript. All authors took part in obtaining relevant accurate information and contributed to the final manuscript through editing and fact-checking.

## Conflict of Interest

The authors declare that the research was conducted in the absence of any commercial or financial relationships that could be construed as a potential conflict of interest.

## Publisher's Note

All claims expressed in this article are solely those of the authors and do not necessarily represent those of their affiliated organizations, or those of the publisher, the editors and the reviewers. Any product that may be evaluated in this article, or claim that may be made by its manufacturer, is not guaranteed or endorsed by the publisher.
